# BRT: An Efficient and Scalable Blockchain-Based Revocation Transparency System for TLS Connections

**DOI:** 10.3390/s23218816

**Published:** 2023-10-30

**Authors:** Qianqian Xing, Xiaofeng Wang, Xinyue Xu, Jiaqi Lin, Fei Wang, Cui Li, Baosheng Wang

**Affiliations:** 1 College of Computer, National University of Defense Technology, Changsha 410073, China; xingqianqian12@nudt.edu.cn (Q.X.); lc_licui17@nudt.edu.cn (C.L.); bswang@nudt.edu.cn (B.W.); 2 Institute of System Engineering AMS PLA, Beijing 100039, China

**Keywords:** PKI and TLS security, revocation, blockchain

## Abstract

Log-based public key infrastructure(PKI) refers to a robust class of CA-attack-resilient PKI that enhance transparency and accountability in the certificate revocation and issuance process by compelling certificate authorities (CAs) to submit revocations to publicly and verifiably accessible logs. However, log-based PKIs suffer from a reliance on centralized and consistent sources of information, rendering them susceptible to split-world attacks, and they regrettably fail to provide adequate incentives for recording or monitoring CA behavior. Blockchain-based PKIs address these limitations by enabling decentralized log audits through automated financial incentives. However, they continue to face challenges in developing a scalable revocation mechanism suited for lightweight clients. In this paper, we introduce BRT, a scalable blockchain-based system for certificate and revocation transparency. It serves to log, audit, and validate the status of certificates within the transport layer security (TLS)/secure sockets layer(SSL) PKI domain. We designed an audit-on-chain framework, coupled with an off-chain storage/computation system, to enhance the efficiency of BRT when operating in a blockchain environment. By implementing a blockchain-based prototype, we demonstrate that BRT achieves storage-efficient log recording with a peak compression rate reaching 8%, cost-effective log updates for large-scale certificates, and near-instantaneous revocation checks for users.

## 1. Introduction

Certificates and CA in TLS/SSL protocol are widely applied to authenticate the domain of web service in web-based client-server systems. It is easy to validate the signature in the certificate structure, so forged and fake certificates provisioning malicious domains can be detected easily. However, the browser cannot easily detect malicious websites whose domain certificates were mistakenly issued by a compromised CA. The CA private key compromise or domain private key compromise need to be addressed. Real-world attacks and failures [1] have occurred where CAs were compromised or misconfigured to issue unauthorized certificates for malicious domains [2,3,4]. In 2020, a significant number of SSL/TLS certificates (over 10,000) were revoked due to key compromise (https://abdalslam.com/ssl-tls-certificates-statistics, accessed on 26 October 2023). The existing CAs’ vulnerability [5] has demonstrated that such failures can further be exploited by adversaries to mount man-in-the-middle (MitM) attacks [6,7].

Therefore, reducing trust in CAs is a topic in the current SSL/TLS PKI. A promising approach, mitigating security issues and threats, is recording the certification operation in a public repository such as Certificate Transparency (CT) [8] and Revocation Transparency (RT) [9]. Log-based PKIs [10,11,12,13], Refs. [14,15] make the revocation and issuance process transparent and accountable, as CAs are obligated to submit revocations to public and verifiable logs. However, log-based PKIs (1) require a centralized, consistent source of information to operate securely, (2) are vulnerable to split-world attacks if the adversary is capable of showing different views of the log to the targeted victims, and (3) do not sufficiently incentivize recording or monitoring CA behavior, unfortunately.

By utilizing blockchain to automate decentralized incentives, IKP [16] contributes a way to audit the conventional PKI. It aims for an audit and punish mechanism to manage the current PKI. However, IKP neither considers certificate chains explicitly nor supports the revocation of a CA certificate, which is unable to defend itself from an attack from the compromised certificate authorities. Moreover, blockchain-based PKIs still lack a scalable revocation mechanism faced with the lightweight client up to now.

Efficient certificate revocation tests have been increasingly paid attention by CRLite [17], Let’s Revoke [18], shared value tree (SVT) [19], TinyCR [20], V’CER [21], and TAP [22]. They employ a variety of compact data structures such as cascaded bloom filter and Othello [23] to efficiently check and update valid certificates and revoked certificates for all TLS connections in an IoT constrained network. However, they lack a mechanism to audit whether the structured certificate log complies with the real world.

It is a crucial challenge to eliminate centralized and vulnerable log servers, meanwhile, decreasing expensive computation around the blockchain. We focus on overcoming the drawbacks of all the proposals above and design a permissionless blockchain-based scalable revocation transparency (BRT) system to enhance the SSL PKI, which efficiently handles events such as certificate transparency proof and monitoring, certificate revocations, and updates. Our smart contract is able to validate scalable proofs of extension of the log (that is, the log is only ever appended) and permit the update of the audit Merkle Tree. Meanwhile, instead of storing and updating the certificate Merkle Tree and CRLs directly in the blockchain, we design a filter cascade to store the status of valid and expired certificates, which is a sequence of compact, probabilistic data structures (e.g., Bloom filters) without either false positives or negatives. Anyone can submit an updated Merkle tree or a filter cascade and help to issue proofs of currency of any given certificate. In summary, we make the following contributions:

- We designed a feasible blockchain-based revocation and certificate transparency framework to enhance the SSL PKI. By utilizing the tamper-resistant, accountable audit and incentive on-chain, the framework eliminates vulnerable centralized servers to log the status of a certificate and operates automatically against split-world attacks. By deploying BRT in conjunction with IKP, our system automatically responds to CA misbehavior with the incentivized monitor and report.
- We designed an audit-on-chain and storage/computation-off-chain mechanism. By rewarding the updating computation off-chain, the smart contract only validates and authorizes a submitted legitimate certificate Merkle Tree, and only publishes the newest address on-chain. Then, the clients exploit the public and authorized log to audit the accountability of certificates.
- We aggregate the revocation information in a cuckoo filter cascade for all certificates that are registered on the blockchain. By optimizing the revocation storage and checking computation, the filters provide browsers with a precise mapping of all certificates to their revocation status and can be easily integrated into modern browsers on the client side since clients only need to download filters and use them to check for revocations of the observed certificates.

The rest of this paper is organized as follows. We review the state-of-the-art approaches for log-based certificate management in Section 2 and give some background in Section 3. We present an overview of the BRT system and the threat model in Section 4. The data structure and the optimization methods are illustrated in Section 5, and the system overview of BRT is shown in Section 6. The detailed design of BRT is shown in Section 7. We present the experimental results of BRT in Section 8 and conclude the paper in Section 9.

## 2. Related Works

### 2.1. Revocation in PKI

CRL and OCSP are the initial attempts to tackle the issue of revocation in SSL PKI. CAs publish CRLs at distribution points, while in OCSP, clients communicate with a CA to obtain the certificate status. However, concerns regarding latency (contacting third parties for revocation checks results in increased connection latencies) and bandwidth (some CRLs exceed 70 MB) have faced criticism (https://www.maikel.pro/blog/current-state-certificate-revocation-crls-ocsp/, accessed on 15 January 2016). Additionally, privacy risks (OCSP checks expose the user’s browsing behavior) and ambiguity (CRL and OCSP servers may experience temporary unavailability due to network errors or active attacks) pose security concerns.

CRLite [17] employs a logically centralized aggregator constructed with Bloom filters, responsible for acquiring all certificates (e.g., from CT logs) and their associated revocation information. However, such aggregators can behave maliciously by incorrectly stating that a valid certificate is revoked, or vice versa. To address the trust issue, they need either to require third-party audits to copy all CRLs and OCSP responses or involve active participation from CAs. However, these options may compromise overall security, as the additional involvement of audit servers or CAs introduces potential vulnerabilities.

### 2.2. Revocation Transparent

To enhance the transparency of the current PKI ecosystem, it is necessary to log certificate revocations. Certificate transparent auditing is a method that can mitigate security threats to SSL PKI certificates by recording certificate operations in a public repository [24,25]. Revocation Transparency [9], proposed as an augmentation to Certificate Transparency, was the initial attempt to facilitate this feature. However, the introduced data structure in Revocation Transparency may result in inefficiencies, since the process of checking revocations remains directly proportional to the number of issued certificates. Furthermore, Revocation Transparency lacks a comprehensive description when checking the revocation status of certificates.

Log-based public key infrastructure approaches, such as AKI [10], ECT [11], ARPKI [12,13], PoliCert [14], DTKI [15], and Coniks [26], prioritize the transparency of certificate issuances and revocations. They all aim to make the revocation and issuance process transparent and accountable, as CAs are obligated to submit revocations to public and verifiable logs. However, we observe that there are still several critical issues in the existing log-based misbehavior monitor schemes [16,27]. Log-based accountable and trustworthy revocation methods, such as CIRT [11], AKI [10], PoliCert [14], ARPKI [12,13], and PKISN [28], aim to replace the existing public key infrastructure (PKI) with a new hierarchical approach that avoids centralizing trust and supports seamless revocation. For example, PKISN [28] addresses the challenge of revoking large numbers of certificates. While it suggests pushing all revocations to clients, it does not provide a solution for encoding the data in a size that is manageable for client. Moreover, the future adoption of these techniques is uncertain due to the requirement of changes to certification authorities (CAs), clients, and, in some cases, certificates.

The deployment of log-based PKI (1) necessitates changes to clients and (in some cases) certificates for policy expression and proof validation; (2) most of them have no scalable ways to push all the accountable TLS certificate revocations to browsers; (3) they require a centralized, consistent source of information to operate securely, and the introduction of log servers enlarges the attack surface; (4) they do not sufficiently incentivize recording or monitoring CA behavior, unfortunately.

### 2.3. Blockchain-Based PKI

Blockchain-based PKI [29,30,31,32,33] exploits the decentralized trust property of blockchain to substitute for conventional PKIs and build new PKI architectures where no CA exists. For example, in Certcoin [15], the basic PKI operations are defined such as registering an identity with a public key or looking up, verifying, and revoking a public key for a given identity. However, there is no identity verification, and whoever first claims the ownership of an identity owns it. Consequently, in the real world, identities (in particular TLS clients) can easily be deceived. Wang [34] and Certledge [35] created their own global certificate blockchains to certificate issuance and revocation transparency. However, they are inefficient in terms of storage costs and impose a heavy scalability pressure on the blockchain due to the following design considerations. (1) A TLS certificate is directly added in certificate transactions to the blockchain periodically during its lifetime. (2) A certificate revocation list(CRL) is added to the blockchain for each revoked certificate. (3) Wang’s proposal has large size headers that comprise DNS names existing in the transactions of the block.

A series of works focus on extending the verification of the PKI certificate log on the blockchain. EthIKS [36] and Trusternity [37] extend Coniks [26] by implementing the transparency log server on the Ethereum blockchain. As their operation cost increases proportionally with the number of users and due to the significant increase in the price of ETH, the system does not scale to large key servers with millions of users.

A blockchain-based accountable structured certificate log method has been proposed [38,39]. CertChain [40] utilizes a dual counting bloom filter (DCBF) with the elimination of false positives. They directly store bloom filters and revocation information in a public blockchain, and this is expensive. ScalaCert [41] proposed revocation-on-chain by employing a redactable consortium blockchain.

Instead of being a new PKI, blockchain-based PKI enhancement schemes such as IKP [16] aim for a check and punishing mechanism to manage certificates in the current TLS/SSL PKI and automatically respond to CA misbehavior. IKP automates responses to unauthorized certificates and provides incentives for CAs to behave correctly. However, this scheme neither provides a certificate public audit nor handles certificate-revocation checking. Moreover, since it neither considers certificate chains explicitly nor supports the revocation of CA certificates, IKP cannot handle the attacks from the compromised certificate authorities as well.

As shown in Table 1, our scheme has dominant advantages over other log-based schemes. Our system provides (1) manipulation-resistance, resilience to split-world attacks, (2) incentivized log monitoring and misbehavior reports, (3) a cost-effective revocation update mechanism, and (4) efficient and scalable certificate status validation for users. We require no changes to TLS or certificates; we only need CA to instantiate a CA entity in our smart contract in the public Ethereum blockchain and submit the issuance and revocation operation to the smart contract straightforwardly. In BRT, we need not record certificates directly. Additionally, the append-only, public, auditable transparency logs and revocation information are recorded out-of-blockchain. We give the design of BRT to address five challenges:

1. Deployability. (1) The blockchain-based component of BRT is implemented on one of the global, public, and permissionless blockchains and is easy to deploy. (2) BRT requires no changes to certificates or TLS, incurs minimal changes to clients, and is incrementally deployable. (3) Any light client node without a blockchain wallet also benefits from BRT, who only needs a little information to push for proof of transparency.
2. Scalability. (1) No certificates are stored on blockchain. (2) Most of the updating computation is kept off-chain. (3) The size of the aggregated revocation information is kept nearly constant.
3. Efficiency. (1) BRT achieves the incremental update of the log-tree efficiently. (2) The computation of a certificate’s proof of transparency is logarithmic to the whole certificates. (3) The revocation maintains a constant time to look up.
4. Incentive. We incentivize monitors by rewarding prompt reports to minimize the attack duration.
5. Privacy. BRT preserves the privacy of the TLS clients, since the browser periodically downloads the compacted log data, and BRT does not know the website information accessed by the browser.

## 3. Background

### 3.1. CA Certificate Attack

In TLS/SSL systems, detecting counterfeit certificates issued to malicious domains is relatively straightforward. However, the browser is less effective at detecting certificates that were mistakenly issued by incorrect or compromised CAs. Such situations often occur due to the leakage of CA or domain private keys. Furthermore, numerous real-world instances have demonstrated this vulnerability [5], where unauthorized certificates were issued to malicious domains [2,3,4] due to misconfiguration. Attackers can further exploit this CA malfunction to launch a man-in-the-middle (MitM) attack [6,7]. The misidentification of the certificate status results from three types of miss-check: The misidentification of the certificate status results from three types of miss-checks: (1) Is the certificate generation trustworthy? (2) Is the certificate revocation trustworthy? (3) Is it possible to continue using a certificate that is in an invalid state?

On one hand, it is crucial to address adversarial events such as CA or domain private key compromises. On the other hand, legitimate actions such as re-creating key pairs and certificates after private-key loss may be mistaken for impersonation attempts. Additionally, switching to a new CA to discontinue using a compromised CA that signs fraudulent certificates may also be misjudged as a malicious event.

### 3.2. Blockchain and Smart Contract

Blockchains, as decentralized peer-to-peer systems, facilitate the implementation of a trustless shared public append-only transaction ledger. This ledger securely records transactions using asymmetric cryptography. To enhance efficiency, transactions are grouped into blocks and subsequently arranged in the blockchain. Peers verify individual transactions by validating cryptographic signatures, while a consensus algorithm determines the transaction order within the network.

The introduction of Ethereum brought about significant advancements in the blockchain technology by incorporating a Turing-complete and stateful programming language. This allowed for the execution of complex code without the need to trust a central party or server. Instead, trust is established through the validation of each program execution on every peer in the network, ultimately reaching a consensus on the outcome. This concept is described as a “world computer” since it effectively operates as a global state machine with user-programmable state transition functions.

Smart contracts, the programs executed on Ethereum, define the system’s state transition function. They are executed in a trustless and tamper-proof manner across the network. However, in order to ensure consistency, Smart Contracts must be deterministic. Peer disagreement on the results of valid executions could lead to an inconsistent state. To maintain liveness in the network and prevent issues such as infinite loops or long-running transactions, Ethereum implemented the concept of “gas”.

Gas assigns a cost to every operation, and an initial endowment is specified for the invocation of a smart contract function. As the endowment is progressively consumed during the execution, it guarantees that even infinite loops eventually halt. Additionally, a block gas limit is set, which determines the maximum complexity of operations a node has to execute in order to validate a block. This limit also establishes an upper bound for the complexity of operations that can be performed atomically on the blockchain.

### 3.3. Cuckoo Filter

Cuckoo filters [42] are used for high-speed set membership tests. It can replace both counting and traditional Bloom filters with three major advantages: (1) it supports adding and removing items dynamically; (2) it achieves higher lookup performance; and (3) it requires less space than a space-optimized Bloom filter when the target false positive rate is less than 3%.

**Figure 1:**
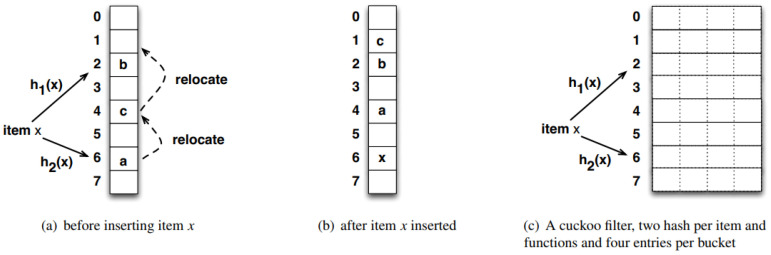
Cuckoo Filter.

The cuckoo filter is a hash index data structure for high-speed member retrieval. The principle is shown in Figure 1, where each member(Item *x*) uses two hash functions $h_{1}\left(x\right)$ and $h_{2}\left(x\right)$ for position (bucket) mapping. The two mapping positions are alternative to each other. If the two mapping positions are empty, choose any fingerprint where the member element is added. If both alternative positions (position 2 and position 6) are occupied as shown in Figure 1a, choose any one to remove the original fingerprint (Item *a*) and place it in your own fingerprint. The removed fingerprint selects your own alternative positionposition 4 to place it in. If the mapping position is occupied again (by Item *c*), as shown in Figure 1b, we repeat the previous process until no fingerprints are removed, otherwise the insertion fails. To reduce the probability of failure, the cuckoo filter can be extended to four slots, as shown in Figure 1c, and a shift operation only occurs when each slot of the bucket to be inserted is occupied.

## 4. System and Threat Models

This section gives a high-level picture of the overall system and introduces the entities involved and basic terminology. We built BRT using Ethereum and the Interplanetary File System (IPFS) [43]. IPFS is a distributed, peer-to-peer file-sharing network that is well-positioned to become the underpinning of a new, decentralized web. Furthermore, it can be accessed by web browsers off-chain directly and by Ethereum smart contracts on-chain by the Provable Oracle service [44].

### 4.1. System Model

**Figure 2:**
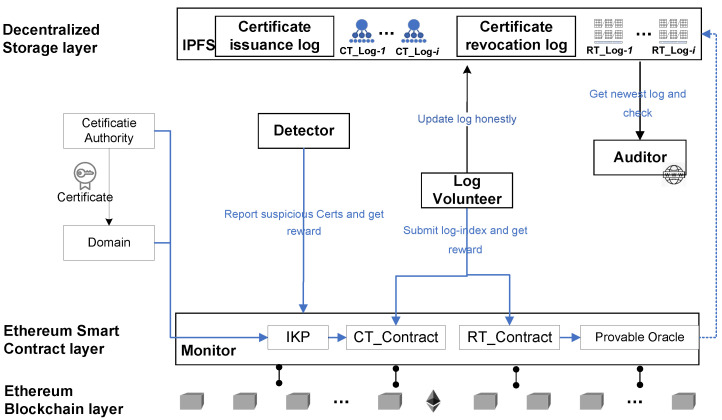
BRT framework.

As shown in Figure 2, our certificate issuance audit framework encompasses six distinct entities: certificate issuance logs, certificate revocation logs, monitors, log update volunteers, detectors, and auditors. Other entities possess similar capabilities and implementation methods as the original certificate transparency scheme.

- Certificate issuance log: All certificates issued by CAs are stored in an associated log structure. These log structures are built using authenticated Merkle hash trees by volunteer log maintainers and are stored on IPFS.
- Certificate revocation log: All unexpired but revoked certificates are stored in an associated compact log data structure. These log structures are built using authenticated cuckoo filter cascades by volunteer log maintainers and are stored on IPFS.
- Monitor: A monitor is a public blockchain contract capable of detecting abnormal certificates and automatically initiating punitive measures against the certificate issuer by sending transactions through the detector. The monitor provides blockchain event logs or query interfaces to assist anyone in verifying the legal visibility of a recorded certificate. Both the issuing authority and domain owner can utilize the monitor to prove and validate the certificate’s legitimacy.
- Log update volunteers: CTLogContract incentivizes volunteers to actively generate the most up-to-date certificate issuance log based on certificate registration events on the blockchain. They store this log in a decentralized database and submit the storage address to CTLogContract in exchange for rewards. Volunteers receive rewards upon the successful verification of their newly submitted log, while CTLogContract ensures the trusted updating of the log.
- Detectors: Public individuals are responsible for voluntarily searching public certificate issuance logs to uncover suspicious or unauthorized certificates. By reporting anomalies and promptly sending transactions to the monitors, they can both constrain the malicious behavior of certificate issuers whose keys are compromised or recently leaked, earning rewards in the process.
- Auditor: Browser TLS clients or independent services can verify whether a specific certificate is recorded in the log through certificate existence proof. This auxiliary function is also provided by the monitor.

### 4.2. Threat Model

We assume there is an active attacker who is able to compromise a CA private key to sign fraudulent certificates, and manipulate a victim’s web traffic, e.g., via a man-in-the-middle (MitM) attack or blocking traffic. An attack against BRT seeks at least one of four outcomes: (1) to make a malicious and fraudulent certificate appear valid, (2) to make a valid certificate appear revoked, (3) to make a revoked certificate to appear valid, or (4) halting a user from obtaining the revocation information.

Everyone is untrustworthy except the CA, and domain entities bootstrapped trust by root CAs in BRT. Every CA honestly submits its certificate issuance and revocation information for its own benefits. We assume that the adversary cannot break standard cryptographic primitives, such as finding hash collisions or forging digital signatures. The adversary also cannot compromise the private keys of arbitrary domains. Given that our solution leverages a blockchain, we also assume that the adversary cannot control a majority of the hashing power in the blockchain network.

## 5. Compact Data Structure Design for Certificate and Revocation Transparency Log

For the practical transparency log auditing, our design exploits the Merkle Tree to record the issuance issue and the cuckoo filter to look up the revoked membership. Merkle hash trees have proof of logarithmic size in the number of certificates/domain owners. For the mass SSL/TLS certificates, keeping the proof of existence only logarithmic to the amount of all certificates is an efficient way to give a certificate transparent issuance and validity. To facilitate the audit of certificate issuance, we outline the log records and proof of existence methods in Section 5.1. Additionally, in Section 5.2, we outline the log records and efficient query methods for certificate revocation, enabling the reliable publication of certificate revocation information.

### 5.1. Binary Merkle Tree for Certificate Transparency Log

**Figure 3:**
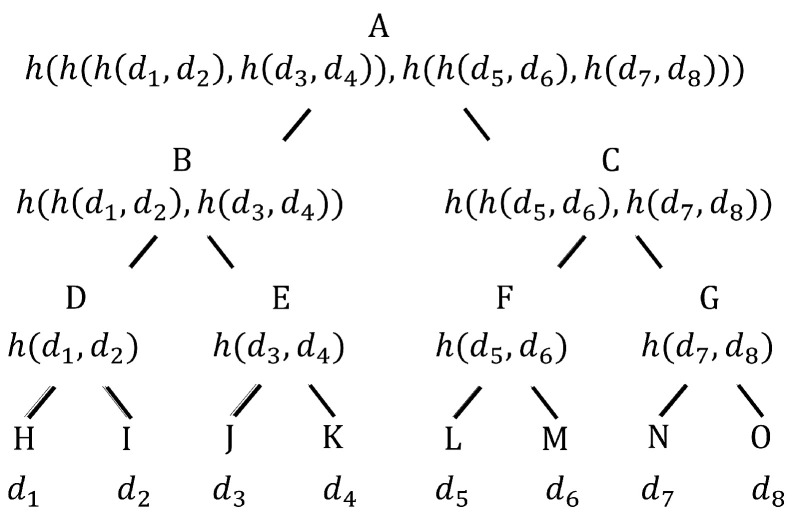
Merkle Tree. *h* represents the collision-resistant hash function, and $d_{1},d_{2},\ldots ,d_{8}$ mean the data items.

A Merkle Tree, also known as a binary hash tree, is a data structure used for efficiently summarizing and verifying the integrity of large sets of data. The Merkle Tree of BRT is mainly used to build transparency log records for certificate issuance. It can provide proof of the existence of any legal certificate. An example of a Merkle Tree is shown in Figure 3, where each leaf node stores a data item $d_{i}$ representing its label. We use the collision-resistant hash function *h* to label the intermediate nodes of the tree by computing the hash algorithm $h(x_{l},x_{r})$, where $x_{l}$ is the label of the left child node and $x_{r}$ is the label of the right child node. The label of the intermediate node *v* is a hash output function that takes the label of the child node of *v* as input.

As shown in Figure 3, the data items {$d_{1},d_{2},\ldots ,d_{8}$} are stored on the leaf nodes, and the hash function *h* is used to calculate the labels of the intermediate nodes, which are calculated from bottom to top to the root node *A*. The hash value stored by *A*, i.e., the root summary, is publicly disclosed. The proof of the existence of the data item *d* in the tree includes data item *d* and the sibling node label of the node in the associated path from the leaf node to the root node. For example, the proof that $d_{3}$ exists in a tree includes {$d_{3}$, ($d_{4}$, $l_{D}$, $l_{C}$)}, where $d_{4}$, $l_{D}$, and $l_{C}$ are labels for nodes *K*, *D*, and *C*, respectively. Given such a proof, the validator calculates the hash value of the root. If the computed hash value matches the common root, the validator output is accepted. Otherwise, the output is rejected. The size of the proof is logarithmic relative to the number of data items stored in the leaf nodes of the tree. Due to the collision-resistance nature of *h*, it is impossible to add new data items to the Merkle hash tree without changing the root digest of the tree, except for the probability that can be ignored.

**Figure 4:**
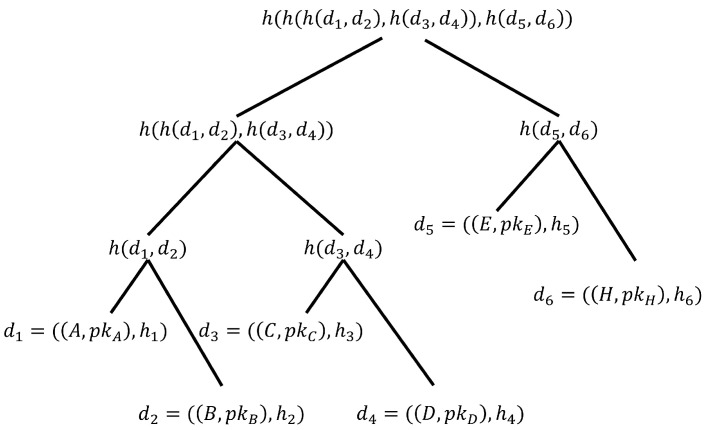
Initial state of the certificate Merkle tree.

MT_CreatTree ($S_{chash}$): Given an initial set of valid certificates of size *n*, the certificate hashes in the set are sequentially used as leaf nodes of the Merkle tree. We instantiate the label of leaf node as $d_{i}=\left((x_{i},pk_{x_{i}}),h_{i}\right)$. Furthermore, the parent node is generated layer by layer according to the Merkle tree generation method shown in Figure 4, until the root node has a root hash. If the set size *n* does not meet the integer power of 2, it must be added by *m* values that are all the integer power of 2. Therefore, *m* full-binary sub-Merkle-trees can be merged from large to small, and the root hash value of the sub tree can be hashed layer by layer to the root of the entire Merkle tree.

**Figure 5:**
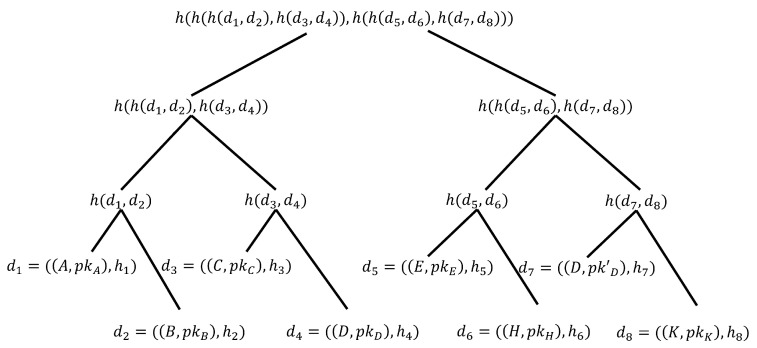
Updating certificate Merkle tree.

MT_UpdateTreeRoot (ΔS, addc): As shown in Figure 5, when a new legal certificate is added, it merges the existing subtrees from small to large until several new unmerged subtrees from large to small are generated, and the root hash value of the subtree is saved. Furthermore, we hash the root hash values of the subtree from small to large until they reach the root of the entire Merkle tree.

### 5.2. Cuckoo Filter Cascades for Revocation Transparency Log

For the practical revocation log auditing, we designed a cuckoo filter cascade to update and look up the revoked membership efficiently and dynamically. Cuckoo filters provide the flexibility to add and remove items dynamically while achieving higher lookup performance and using less space than conventional bloom filters [42,45] for applications that require low false positive rates (<3%).

**Figure 6:**
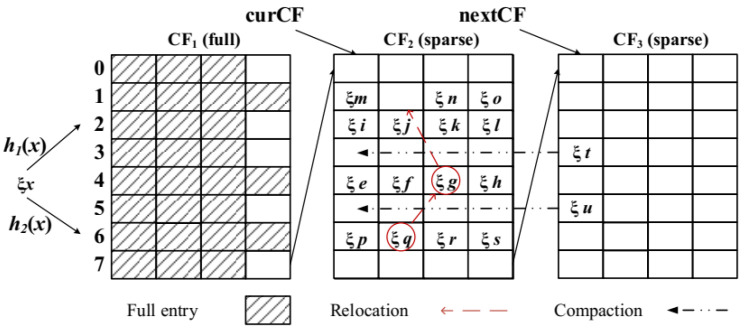
Dynamic Cuckoo Filter.

Although the cuckoo filter can achieve better space utilization than the bloom filter, it still encounters situations where the entire filter cannot be inserted, and a new filter must be constructed when applied to dynamic massive data indexing. The dynamic cuckoo filter is designed to alleviate such problems. The dynamic cuckoo filter, as shown in Figure 6, consists of several dynamically increasing and decreasing index entries (CF) forming an item queue (CFQ), which actively adds new index entries to store removed fingerprints when the current index entry fails to store. The main algorithms are as follows:

CF_ Insert (*x*): When inserting a new element *x* into the current index entry curCF, first, calculate its fingerprint ξ = Fingerprint(x) and two alternative positions, $i_{1}=hash(x)$, $i_{2}=i_{1}$⊕hash(ξ). Randomly select $i_{1}$ or $i_{2}$ as bucket number *i* for storage ξ. If the corresponding bucket is full, randomly select one fingerprint to replace it. For the replaced fingerprint ξ, calculate the alternative position i=i⊕ hash(ξ). If there is space in the new bucket location, store it ξ. Otherwise, continue to repeat the previous process until the storage is completed or the conflict fails. The last fingerprint to be replaced is the victim. At this point, store the next entry nextCF or create a new CF as the new current entry curCF until no new conflicts appear.

CF_ Lookup (*x*): Calculate fingerprints for the element *x* to be searched for ξ, and search for positions $i_{1}$ and $i_{2}$ for all CF searches for corresponding positions. If fingerprints are present ξ, then, prove the existence of *x*; otherwise, it does not exist.

CF_ Delete (*x*): Furthermore, for the element *x* to be deleted, first, CF_ Lookup (*x*) finds the storage location and deletes the corresponding fingerprint.

CF_ Compact: For CFQ sparsity caused by deletion operations, regular merging can be performed. The fingerprints in each bucket of the sparse CF can be moved forward from front to back in the queue, and the excess empty CF can be removed from the queue.

An efficient retrieval data structure for revocation publishing not only requires high space utilization and retrieval efficiency but also the ability to dynamically add and delete the increasing number of revoked certificates that have expired. More importantly, the accuracy should be close to 100%, and there should be no false positives. Therefore, simply using filters cannot meet the requirement of zero false positives. Therefore, we extend the use of a new data structure by cascading two layers of CFQ with bloom filters. For the legitimate certificates that cause false positive results in the first level of CFQ, we add the second level of CFQ as a whitelist. For the revocation certificates that cause false positive results in the second level of the filter, we add the third level of the bloom filter as a blacklist.

**Figure 7:**
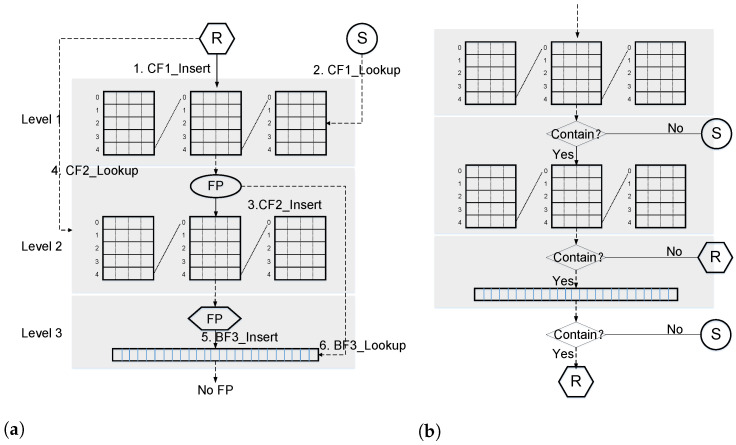
Cascaded Cuckoo Filter for Certificate. (**a**) Cascaded Cuckoo Filter Element Insertion, (**b**) Cascaded Cuckoo Filter Element Retrieval.

The cascade cuckoo filter (CCF) we designed is shown in Figure 7, assuming that the cascade filter has a total of three stages, with different hash functions used for the first two CFQs. The set of unexpired certificates is U=R∪S, where the revocation certificate set is *R*, the valid certificate set is *S*, and R∩S = ⊘.

CCF_ Init (*R*, *S*): Initializing CCF mainly includes creating cascaded filters, adding revocation certificate sets, and checking valid certificate sets. First, perform CF1_ Insert (*x*) operation on each element x∈R. Next, perform CF1_ Lookup (*y*) on each element y∈S of the valid certificate set, which forms a set FP1 of all valid certificates with existing search results. For each element y∈FP1, perform the CF2_ Insert (*y*) operation to add it to the second level CFQ. Next, for each element x∈R, perform the CF2_ Lookup (*x*) operation, which forms a set FP2 of all revocation certificates with the existing retrieval results. For each element x∈FP2, perform the BF3_ Insert (*x*) operation to add it to the third level bloom filter. Then, for each y∈FP1, perform the BF3_Lookup (*y*) operation. If none exists, then end this process. Otherwise, repeat the above process.

CCF_ AddRevoc (ΔR): When a new set of revocation certificates ΔR arrives, the revocation set is $R^{\prime }=R\cup \Delta R$, and the valid certificate set is $S^{\prime }$ = *S*/ΔR. Perform CF1_ Insert (*x*) for each element x∈ΔR operation. Compare the first level CF fingerprints of ΔR and $S^{\prime }$ to obtain the set cfm1 where the fingerprints overlap. For z∈cfm1, perform CF1_ Lookup (*z*); if the search exists, run CF2_ Insert (*z*). Perform CF2_ Lookup (*x*) for each element x∈ΔR operation; if the search exists, run BF3_ Insert (*x*).

CCF_ Lookup (*x*): As shown in Figure 7b, for any legally issued but unexpired certificate *x*, first, retrieve it through the first layer filter, i.e., CF1_ Lookup (*x*). If it is not retrieved, it is proven to be a valid certificate. Otherwise, perform a second layer filter search, i.e., CF2_ Lookup (*x*); if it is not retrieved, it is proven to be a revocation of the certificate. Otherwise, perform a third layer filter search, i.e., BF3_ Lookup (*x*), if it is not retrieved, it is proven to be a valid certificate; otherwise, the certificate is revoked.

CCF_ Delete (ΔR): For all x∈ΔD, only CF1_ Delete (*x*) is performed, as the third layer cascade filter is a bloom filter that cannot be deleted.

CCF_ UpdateCert (ΔS): Perform CF1_ Lookup (*y*) first for all y∈ΔS; if search exists, perform CF2_ Insert (*y*). Compare the second level CF fingerprints of *R* and ΔS to obtain the set of overlapping fingerprints cfm2. For z∈cfm2, perform CF2_ Lookup (*z*); if search exists, perform BF3_ Insert (*z*).

CCF has several advantages over the classic bloom filter: (1) it supports the dynamic addition and subtraction of elements, (2) it supports more efficient storage and querying (even when the space occupancy rate reaches 95%), and (3) it uses actual smaller space storage when the false positive rate is less than 3%.

## 6. Blockchain-Based Certificate and Revocation Transparency Overview

We built BRT on the basic framework of IKP [16]. Our BRT framework establishes a comprehensive and automated certificate audit system capable of conducting audits with minimal supervision. It also automatically carries out response strategies, such as rewards and punishments, upon the detection of malicious behavior reports. Furthermore, this contract governs the transmission and reception of payments through a global fund and serves as a trustee intermediary responsible for managing funds and making payments to all other parties.

### 6.1. Smart Contract as a Trustworthy Audit and Publish Service

#### 6.1.1. Permissioned vs. Permissionless

Most of the state-of-the-art blockchain-based transparency systems utilize the block- chain solely as a ledger for TLS Certificates and their revocation status. Storage-on-chain is a challenge to blockchain due to the increasing number of certificates. Moreover, full nodes need to keep an updating copy of log events from the blockchain to maintain the status of certificates as up-to-date. Alternatively, light nodes need to send transactions to query the certificate status, which results in increased communication latency. Due to the limited scalability of permissionless blockchains, the latency may surpass that of conventional approaches, given the high volume of SSL connections per second on the Internet. Even though exploiting a permission blockchain is a good choice to handle the blockchain scalability, less security and less accessibility are obstacles to it taking its place in the field of B2C applications.

#### 6.1.2. Scaling by Off-Chain

We propose deploying our system on the Ethereum platform using an off-chain scaling solution to enhance scalability. Since the audit smart contract in our BRT contains a significant amount of data and computational requirements, we have devised an audit-on-chain, store/computation-off-chain framework to ensure certificate and revocation transparency. To access the audit log stored in a distributed file system, we employ a data carrier oracle. Additionally, we connect two computation oracles to the computation data sources, enabling auditable execution of the two scripts involved in updating the audit log and revocations in BRT. This approach allows the audit smart contract to execute external program routines in a trustless manner. Our off-chain computation solution not only alleviates the burden on the Ethereum blockchain but also reduces the cost associated with transaction verification and processing.

#### 6.1.3. Timestamping for Revocation Policy

It is widely recognized that revoking a private key owned by an important certificate authority (CA) should invalidate all certificates signed with that key. This is crucial as we cannot determine if these certificates were fraudulently created. However, if a compromised CA can determine the exact time of the attack or when an illegitimate action was first observed, we can ensure that all certificates signed before the attack are still considered valid. Thus, to prevent collateral damage resulting from the revocation of CA certificates, a trusted timestamping service is needed.

In the case of BRT, this service is implemented using smart contracts. Depending on the deployment scenario, either a domain or a CA submits the certificate to a log. Each new certificate is appended to the log along with its registration timestamp. Subsequently, anyone who has access to the commitment can query the log to obtain a presence proof for a specific certificate. This presence proof includes the registration timestamp, serving as evidence that the log contained the given certificate at a specific point in time. As a result, a requester can confidently assert that the certificate was generated before the provided timestamp.

### 6.2. IKP as the Automatic Audit Response Framework

IKP [16] mainly implements a PKI automatic audit response framework, with the goal of providing an incentivized game environment where PKI certificate behaviors of all parties are recorded and monitored, achieving an automatic response to malicious behavior.

That is to say, BRT uses the Ethereum platform to incentivize CAs to issue certificates correctly through economic means, introduces motivated testers to report illegal certificates, and imposes economic penalties on CAs who issue illegal certificates. The participants in the BRT system include three participants interacting in the TLS protocol system, namely, domain, CA, and client, and the other important role, the detector who reports suspicious certificates. For CA, this means issuing unauthorized certificates without being penalized or being able to accept more payments than fines. Domains can engage in malicious behavior together with detectors and CAs through collusion attacks, and detectors can attempt to report every certificate they encounter and report it, hoping to report a certificate that is actually unauthorized and receive rewards. Under this framework, the goal of all participants is a positive return on investment (ROI).

There are several PKI certificate trust management operations in the BRT main framework contract to achieve domain and CA-oriented entity creation and update, as well as malicious behavior report inspection and response mechanisms for detectors, including:

- CA Register: CA registers its information with the BRT contract. At the same time, the relevant certificate hash is stored in a temporary data structure in the blockchain contract as a reserve for subsequent certificate issuance log updates. It is worth noting that the root CA did not review the contract address during registration, but the intermediate CA needs to review the contract to specify its parent CA information.
- CA Update: A certain updateAddrs of CA can update CA information. This situation usually occurs when the CA holder’s account layout has leaked the key. To prevent malicious attackers from using it as a domain subscription response policy, it is necessary to replace the CA holder account address layout. The update condition for CA’s public key pubkey is that the corresponding private key of the pubkey is leaked. Currently, a new private key and certificate are used, and the pubkey information is updated.
- Domain Registration (DCP Register): Registering a domain’s own DCP indicates a constraint that allows a CA to authorize its certificate and is used to describe what kind of CA issuance behavior is considered a non-compliant error behavior. The contract can use the DCP published by the domain to automatically check the certificates reported by the detector to ensure that legitimate certificates are issued in accordance with the domain certificate policy. DCP can provide policy expression capabilities such as CA whitelist and short-term certificate enforcement.
- Domain Update (DCP Update): Similar to the CA update, a certain updateAddrs in the domain can update DCP information. The domain updates the review policy through DCP update, which allows for the flexible selection of different certificate policies by changing the review contract.
- RP Negotiation: CA first registers RP in the BRT main framework contract. The domain orders these policies from the CA to automatically respond to CA error behavior. The amount paid for the subscription serves as an economic incentive for CA not to issue unauthorized certificates. If an unauthorized certificate is not issued for the domain before the expiration, and the CA’s behavior is compliant, the CA receives this reward. RP can provide responses such as financial expenses and CA revocation. RP serves as insurance for the domain against losses caused by malicious or mis-issued certificates, as if unauthorized certificates of the domain are detected, the domain and the detector receive rewards from RP.
- Misbehavior Report: The detector sends unauthorized certificates (indicating CA misconduct) to the BRT contract to report suspicious certificates. The BRT contract initiates an audit by checking the certificate through the domain’s DCP, and if the certificate is indeed unauthorized, triggers the appropriate RP to respond.

## 7. Details in Blockchain-Based Certificate and Revocation Transparency

**Figure 8:**
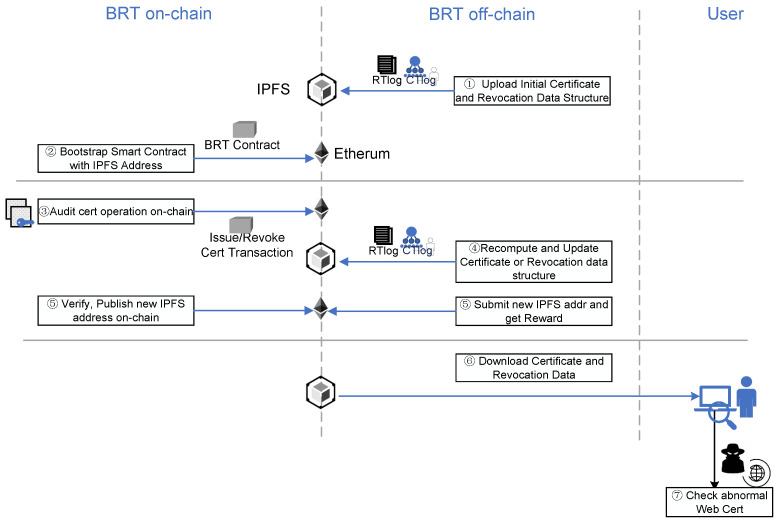
The dataflow and process of BRT.

In this section, we present a specific design for the BRT smart contract. First, we introduce the dataflow and process in our blockchain-based certificate management framework as shown in Figure 8. We utilize the BRT smart contract to handle the issuance and revocation of SSL PKI certificates in real networks, ensuring the creation of public and trustworthy records. The BRT contract holds the legal and trustworthy status for any certificate activity recorded in the blockchain.

1. Pre initialization. We collect the certificate status of the entire network in advance, organize the certificate transparency log and certificate revocation query data structure according to two data structures in Section 5, namely Merkle tree(MT) and cascaded cuckoo filter(CCF), upload them to IPFS, and record the certificate log address of the initial state on IPFS.
2. Bootstrap. In the Bootstrap phase, the system deploys the BRT smart contract set on Ethereum and records the initialization log address at IPFS. Once the IKP contract is started, the CTLogContract contract is also begun to initialize and configure the current log tree certification information. RTLogContract is also deployed on-chain and publishes the index address of the current cascaded cuckoo filter at IPFS.
3. All CAs can record the certificate issuance and revocation operations on the blockchain through Ethereum transactions to the BRT smart contracts. At the same time, detectors can report CA misconduct that does not conform to the certificate policy at any time and submit expired certificates for inspection.
4. Incentive updating log off-chain. Volunteers monitor the certificate operation on the chain at any time, collect information about newly issued certificates and revoked certificates, organize new data structures for certificate transparent logs and certificate revocation records, and actively store the latest logs in IPFS.
5. Audit on-chain. Volunteers send the “submit” transaction to CTLogContract to update the certificate issuance log and ask for a bonus. CTLogContract obtains the root of the submitted Merkle tree on IPFS and then performs the verification calculation according to the newly issued certificate data cached on the chain, updates the certificate log status, and rewards the volunteers. Similarly, volunteers send the “submit” transaction to RTLogContract for certificate revocation log updating and to ask for bonuses. The RTLogContract contract combines the cached data on the chain, the index of the certificate revocation log in the previous state, and the index address of the new certificate revocation log submitted by the volunteers. The verification calculation is started through the Provable Oracle [44]. If the verification is passed, the volunteers are rewarded, and the new index address is recorded on the chain for users to retrieve.
6. User synchronous update. The user receives synchronously the latest certificate log index address published on the chain and downloads the latest certificate log information from IPFS.
7. User certificate check. When a web domain is accessed by the local browser, the user can check the status of this domain certificate.

Overall, our certificate logs are stored and processed off-chain, minimizing the computational burden on blockchain transactions and maximizing scalability.

### 7.1. CTLogContract Operation on Blockchain

In this section, we elaborate on the functions and implementation methods of log update volunteers. These volunteers are motivated by rewards and actively contribute by submitting the most up-to-date certificate issuance log to CTLogContract. The update operations that involve interacting with CTLogContract consist of the following two steps:

CT_ TreeCommit (commithash): CTLogContract incentivizes volunteers who offer out-of-chain log update services. Before claiming the reward for log updates, volunteers hash their updated results, randomly select secret values, and register the hash values in the blockchain, as well as submit a deposit.

CT_ TreeReveal (newroot, secret, ${\mathrm{Tree}}_{\mathrm{index}}$): Volunteers submit the root hash, selected secret value, and IPFS storage address of the new Merkle tree they upload. The contract first verifies the existence of the consensus through the equation consensus=hash(newroot,secret) and, if so, proves that the volunteer is the current submitter. Next, additional calculations are performed to verify the accuracy of the new root hash and whether it is stored at the IPFS storage index address ${\mathrm{Tree}}_{\mathrm{index}}$. If all the above conditions are met, the volunteer receives a reward. Otherwise, the deposit is seized. Additionally, the contract broadcasts the storage address and root hash of the new certificate issuance log tree to the entire network by adding events to on-chain transactions.

In our scheme, the CTLogContract stores only the minimum information necessary for verification and updates. The blockchain does not store all the Merkle tree information; instead, volunteers submit it to the offline IPFS storage system. The blockchain ensures the correctness and credibility of certificate issuance logs at a minimal storage cost. Additionally, it uses a reward mechanism to store trusted certificate issuance logs in an easily accessible decentralized storage system, IPFS, making it easier for domain owners who successfully register certificates to obtain the transparency proof of their own certificates.

### 7.2. RTLogContract Operation on Blockchain

The cascaded cuckoo filter acts as a trusted, compressed, and efficiently searchable log for revocation certificates, providing browsers with an accurate mapping of certificate revocation states. Whenever a new certificate revocation transaction is submitted to the BRT principal contract, the BRT sends a transaction to the RTLogContract contract for caching the revocation certificate hash and publishing a revocation event. After a certain period of time, volunteers collect new certificate issuance and revocation events that occurred during that period and calculate new cascaded filters based on their own knowledge of the state. These cascaded filters are then stored off the blockchain in the decentralized storage system IPFS, and motivated volunteers submit them to IPFS in exchange for rewards from the RTLogContract contract.

The certificate revocation chain comprises four stages: the registration stage for verifying the script for revocation log updates, the application stage for revocation log updates, and the verification stage for revocation log updates. The first stage takes place after the creation of the RTLogContract contract, while the second and third stages must be performed sequentially.

RT_Registerscript (${\mathrm{script}}_{\mathrm{index}}$): The registration process for the validation script of revocation log updates occurs during the initialization phase following the creation of the RTLogContract contract. The verification algorithm for revocation log updates is stored in script form within the decentralized storage system IPFS. The contract maintainer utilizes the address ${\mathrm{script}}_{\mathrm{index}}$ as a parameter to transmit the validation script for revocation log updates and register it as a transaction in the RTLogContract contract.

RT_Validate (prevCCF, R, S, ΔD, ΔR, ΔS, curCCF): The content of the revocation log update validation script is: For the original revocation set R and the legal certificate set S, first, perform CCF_ Delete (ΔD) on the expired certificate, and then, perform CCF_ AddRevoc (ΔR) on the new revoked certificate, and finally, perform CCF_ UpdateCert (ΔS) on the newly added valid certificate, compare the new ${\mathrm{curCCF}}^{\prime }$ obtained with curCCF, and if it is the same, verify that curCCF is indeed the current new cascaded filter. Otherwise, verify that curCCF is an error.

RT_CCFCommit (consensus): The smart contract provides rewards for volunteers who provide out of chain log update services. To claim the reward for log updates, volunteers first hash their updated results and randomly selected secret values, register the hash values in the blockchain, and pay a deposit.

RT_CCFReveal (expcerts, expn, secret, Filter_index): Volunteers submit a set of expn expired certificates they wish to update, expcerts, the selected secret value, and the IPFS storage address Filter_Index they upload. The contract first verifies whether there is Commithash=hash(Filter_index,secret), if any, prove that the volunteer is the current new submitter, and then, further verify through calculation whether the certificate set expcerts are all invalid certificates. If all the above are true, then use oracle to start the trusted computing environment and execute the update verification script. If it is verified that (1) certificates in the set expired are indeed stored in the IPFS at the Filter_index address, (2) if the new revocation log calculation in IPFS at the the Filter_index address submitted by volunteers is correct, the volunteer receives a reward. Otherwise, the deposit is confiscated. Furthermore, the contract notifies the entire network of the storage address Filter_Index for the new certificate revocation log by adding events to the blockchain transactions.

## 8. Evaluation

In this section, we first deploy BRT on the locally built blockchain test network. The BRT smart contract set is composed of three main contracts, and the transaction cost for deploying every contract is evaluated.

As one of the key functions, we evaluate the performance of certificate Merkle tree log update validation on-chain. The storage cost and computation cost on-chain are verified through experiments.

Finally, we focus on the certificate revocation log update validation, starting with selecting more efficient cascade filter parameters and conducting experimental verification and performance evaluation.

### 8.1. BRT Contract Deployment and Cost Assessment

We used Solidity to implement BRT smart contracts, compile and simulate the EVM environment in Remix, and use a browser interface to simulate transactions on smart contracts. We provide, in Table 2, the approximate calculation steps and the converted economic expense for the BRT entity contract, certificate issuance log contract CTLogContract, and some heavy transaction operations that they support.

According to the trading data of Ethereum in October 2023, **1** ether = 1.796,249 dollar (https://www.finanzen.net/devisen/ethereum-dollar-kurs, accessed on 25 October 2023). The gas price is increasing and it is unacceptable to directly spend for the deployment of BRT, so we chose a sidechain testnet named Polygon Mumbai [46] (Other options are cardano [47]). As the Polygon mainnet showed, **1** MATIC is $10^{9}$ Gwei and 0.518 dollar, 1 MED GAS = 51.3 Gwei (https://coincodex.com/crypto/matic-network/, accessed on 25 October 2023). From the table, it can be seen that the economic cost of creating BRT entity contracts is relatively high, but the contract creation only needs to be carried out once, and the gas cost of the subsequent registration and update operations is within an acceptable range according to the economic expense.

### 8.2. Overhead of Certificate Transparent Log Updating On-Chain

We validated the operational cost on-chain, as volunteers submit certificate issuance log to the IPFS repository (https://ipfs.io/ipfs/QmeCaobKXQdDgRj9zto6pxeGJByemCWEKTSFik4qAna9c5, accessed on 25 October 2023) and apply to the CTLogContract contract for updating and receiving a reward.

**Figure 9:**
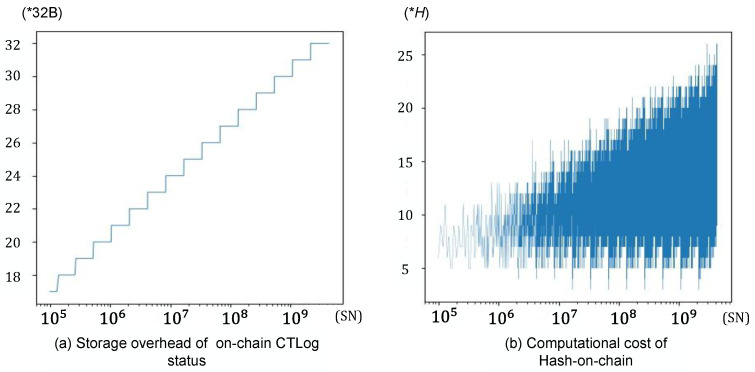
The on-chain cost of the Merkle tree. “*32B” in (a) represents the storage overhead is in units of 32Bytes, and “*H” in (b) means the computational cost is calculated in units of a hash operation.

We first show the relationship between the number of certificates and the size of the storage overhead in CTLogContract in Figure 9a. Since the full-size Merkle tree is stored off-chain, CTLogContract only stores the intermediate cache status of the Merkle tree, which is only for verifying the submitted updating transactions. We use the data item size of 32B to store a node label of the Merkle tree, and CTLogContract is able to use 30 items to handle the log updating for billions of certificates (i.e., $10^{9}$ as shown in Figure 9a). Mentioning that the direct storage of the Merkle tree on-chain for certificate validation is O(nlog(n)), our scheme only cost O(log(n)) storage on-chain.

As shown in Figure 9b, we perform update operations on the CTLog contract at a rate of one certificate per update (in actual scenarios, the updates are not so frequent, and the cumulative computational cost is less). Based on the coordinates, it can be seen that the computation costs of the Merkle tree on-chain in the CTLogContract generated by the update operation increase logarithmically with the number of certificates stored in the log. Our scheme only costs O(log(n)) computation on-chain, which can efficiently accommodate more certificate records. As demonstrated in Figure 9b, CTLogContract only costs 25 times the hash computation to handle the log updating for billions of certificates (i.e., $10^{9}$, as shown in Figure 9b).

### 8.3. Performance of Cascaded Cuckoo Filters

The usage statistics of LetsEncrypt (https://trends.builtwith.com/ssl/LetsEncrypt, accessed on 25 October 2023) show that they have signed almost 55M SSL certificates until this year. The proportion of revoked certificates [48] is maintained at 0.35∼2.4%, and among the most popular CAs, GoDaddy stands out, with 34.5% of its certificates being revoked before expiration. Since it is not easy to obtain legally issued and revoked certificates worldwide in a limited time, we have simply extracted almost 800 K (798,901) certificate samples by the certificate revocation analysis tool (https://github.com/casebenton/certificate-revocation-analysis, accessed on 25 October 2023) to assess and analyze the performance of cascaded cuckoo filters.

**Figure 10:**
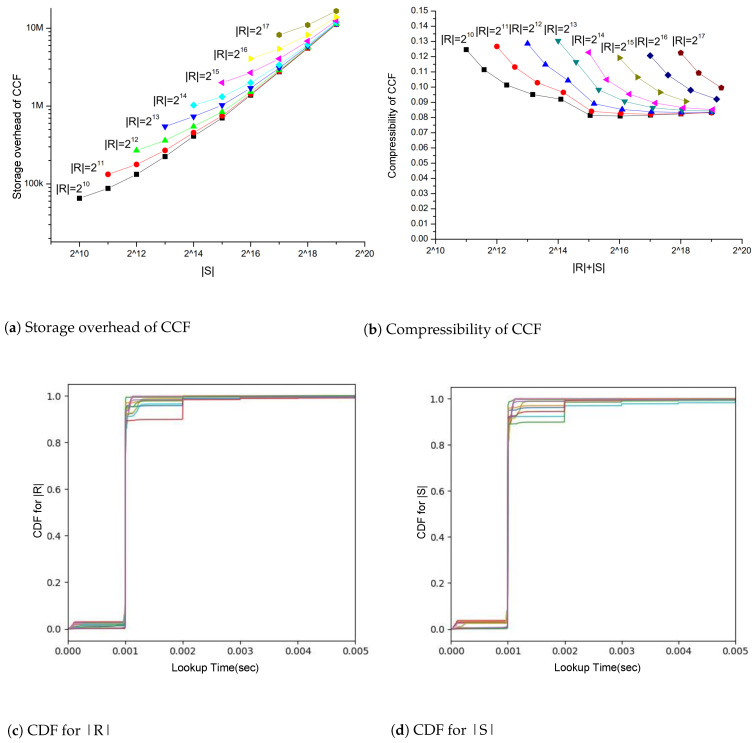
Performance of Cascaded Cuckoo Filter. Storage overhead of Cascaded Cuckoo Filters is in (**a**); Compression rate of Cascaded Cuckoo Filter is in (**b**); Query Performance Distribution with |R| is in (**c**); Query Performance Distribution with |S| is in (**d**). The color lines in (**c**,**d**) represent the different sizes of |R| as the same as in (**a**,**b**).

We set the maximum supported certificate number of our cascaded filters as $2^{20}$ and set capacity = 400,000, bucket_size = 4, fingerprint_size = 1 for the first-layer cuckoo filter, and capacity = 10,000, bucket_size = 4, fingerprint_size = 1 for the second-layer cuckoo filter. Since there are notable exceptions that most CAs have much fewer revoked certificates than non-revoked certificates [48], we limit without loss of generality that the size of valid certificates |S| is not smaller than the size of revoked certificates. As shown in Figure 10a, when the size of valid certificates |S| is $2^{18}$ and the size of revoked certificates |R| is $2^{17}$, we achieve 10 M of CCF storage. The same setting results in a compression ratio of 11%, as shown in Figure 10b. Figure 10a,b demonstrate that the storage cost of cascaded filters exhibits a linear relationship with the overall size of the certificate set, while also maintaining a consistent compression. Especially, the compression ratio for certificate storage ranges between 8% and 14%, with a peak compression rate nearing 8%, as illustrated in Figure 10b.

We tested the query performance of the cascaded cuckoo filter using Intel (R) Core (TM) i5-4300U, 1.90 GHz CPU, and 4G of memory. As shown in Figure 10c,d, regardless of the size of the legitimate certificate set and revocation certificate set used, whether it is the revocation certificate element or legal certificate element, the query time is less than 1 ms for exceeding 99.6% of the elements.

Overall, we employ blockchain as an immutable storage for the necessary proofs during the auditing process. To optimize costs, we not only decrease computations and storages on-chain as much as possible but also give a highly compacted revocation log for users to constant-time and fast look-up malicious revoked certificates. As a result, our scheme guarantees security, ease of implementation, suitability for large-scale key servers, and lightweight performance for clients.

## 9. Conclusions

We propose a blockchain-based TLS certificate manager framework, named BRT, which implements a scalable and easily deployable certificate and revocation transparency method for recording, auditing, and verifying the status of SSL PKI certificates. We have designed an on-chain audit and off-chain storage/computing framework to improve the operational efficiency of BRT on the blockchain. BRT achieves storage-effective log recording with a peak compression rate nearing 8%, cost-effective log updating for large-scale certificates, and almost constant-time revocation check for users. As the experiment has shown, BRT introduces reasonable overheads in term of storage, validation computing, and incentive cost.

Considering that blockchain as a technology has its own restrictions, such as a low throughput, which cause the possibility of a long response time and high transaction fees, there have emerged several solutions to enhance the scalability of blockchain, which have been categorized into two categories [49]. One is building an independent distributed PKI protocol with custom consensus and cryptocurrency economy such as proof-of-stake(PoS) [50], delegated proof-of-stake(DPoS) [51], instead of proof-of-work(PoW) [52]. The other category is building the off-chain channel [53]. Moreover, supporting accountable off-chain computation is the key characteristic to design an efficient and secure application around blockchain. Researchers have focused on the method using decentralization, trusted nodes, premium data, and cryptographic proofs to connect highly accurate and available data/APIs to any smart contract, which can seamless connect to any API off-chain [54,55,56,57] with different oracle tools [58,59,60]. We are already in the process of optimizing BRT by utilizing these tools.

It would be also interesting to apply efficient functional storage proof structures such as AAD [61], Merkle2 [62], and vector commitments [63,64] around blockchain to speed up certificate aggregation and verification times, as well as reduce proof size.

## Figures and Tables

**Table 1:** Comparison of Log-based schemes to Certificate Management.

Schemes	CT [8]	AKI [10]	ARPKI [13]	DTKI [15]	Wang [34]	Certledger [35]	BRT
Resilient to split-world/MITM attack	✘	✘	✘	✘	✘	✔	✔
Built-in revocation transparency	✘	✔	✔	✔	✔	✔	✔
Eliminates client certificate validation	✘	✘	✘	✘	✔	✔	✔
Eliminates trusted key management	✘	✘	✘	✘	✘	✔	✔
Preserves client privacy	✘	✔	✔	✘	✔	✔	✔
Require external auditing	✔	✔	✔	✔	✘	✘	✘
Monitoring promptness	✘	✘	✘	✘	✘	✔	✔
External info collecting during TLS handshake	✔	✔	✔	✔	✔	✘	✘
Logarithmic Time for cert transparency proof	✔	✔	✔	✔	✘	✘	✔
Constant time for revocation look-up	✘	✘	✘	✘	✘	✘	✔
Monitoring incentive	✘	✘	✘	✘	✘	✘	✔
Off-chain update and storage for full logs	-	-	-	-	✘	✘	✔

**Table 2:** BRT Trans cost.

Trans Type	GAS	Economic Expense	Trans Type	GAS	Economic Expense
Register CA	257,555	$0.0684	Register DCP	189,261	$0.0503
Update CA	66,105	$0.0176	Update DCP	37,078	$0.0009
Order RP	176,727	$0.0470	pre-report cert	27,640	$0.0007
LogRegister	65,066	$0.017	Report cert	148,543	$0.0039
DCPCheck	615,319	$0.0164	RPReaction	605,995	$0.0161
cost of BRT main contract bootstrap				6,726,872	$0.1788
cost of CTLog_contract bootstrap				1,670,503	$0.0444
cost of RTLog_constract bootstrap				1,688,567	$0.0449

## Data Availability

Not applicable.
